# Supplementary material for: Peripheral Soluble Immune Checkpoint-Related Proteins Were Associated with Survival and Treatment Efficacy of Osteosarcoma Patients, a Cohort Study
Source: Cancers (Basel). 2024 Apr 24;16(9):1628. doi: 10.3390/cancers16091628 (PMC11083464; doi:10.3390/cancers16091628)
Supplement: Supplementary file 1 [file cancers-16-01628-s001.zip › cancers-2934872-supplementary.pdf]

Supplemental Materials

Peripheral Soluble Immune Checkpoint-Related Proteins Were Associated with Survival and Treatment Efficacy of Osteosarcoma Patients, a Cohort Study

Binghao Li [1,2,†], Qinchuan Wang [3,4,5,*,†], Yihong Luo [4,5], Sicong Wang [4,5], Sai Pan [4,5], Wenting Zhao [4,5], Zhaoming Ye [1,2] and Xifeng Wu [4,5,*]

[1] Department of Orthopedics, Second Affiliated Hospital, Zhejiang University School of Medicine, Hangzhou 310009, China; libinghao@zju.edu.cn (B.L.); yezhaoming@zju.edu.cn (Z.Y.)
[2] Clinical Research Center of Motor System Disease of Zhejiang Province, Hangzhou 310009, China
[3] Department of Surgical Oncology, Affiliated Sir Run Run Shaw Hospital, Zhejiang University School of Medicine, Hangzhou 310016, China
[4] Center for Biostatistics, Bioinformatics and Big Data, The Second Affiliated Hospital and School of Public Health, Zhejiang University School of Medicine, Hangzhou 310009, China; yihongluo@zju.edu.cn (Y.L.); wangsicong@zju.edu.cn (S.W.); ps@zju.edu.cn (S.P.); zwt376@zju.edu.cn (W.Z.)
[5] The Key Laboratory of Intelligent Preventive Medicine of Zhejiang Province, Hangzhou 310058, China
* Correspondence: wangqinchuan@zju.edu.cn (Q.W.); xifengw@zju.edu.cn (X.W.); Tel.: +137-7746-8656 (Q.W.); +171-3248-0685 (X.W.)
† These authors contributed equally to this work.

Supplemental Figures and Tables

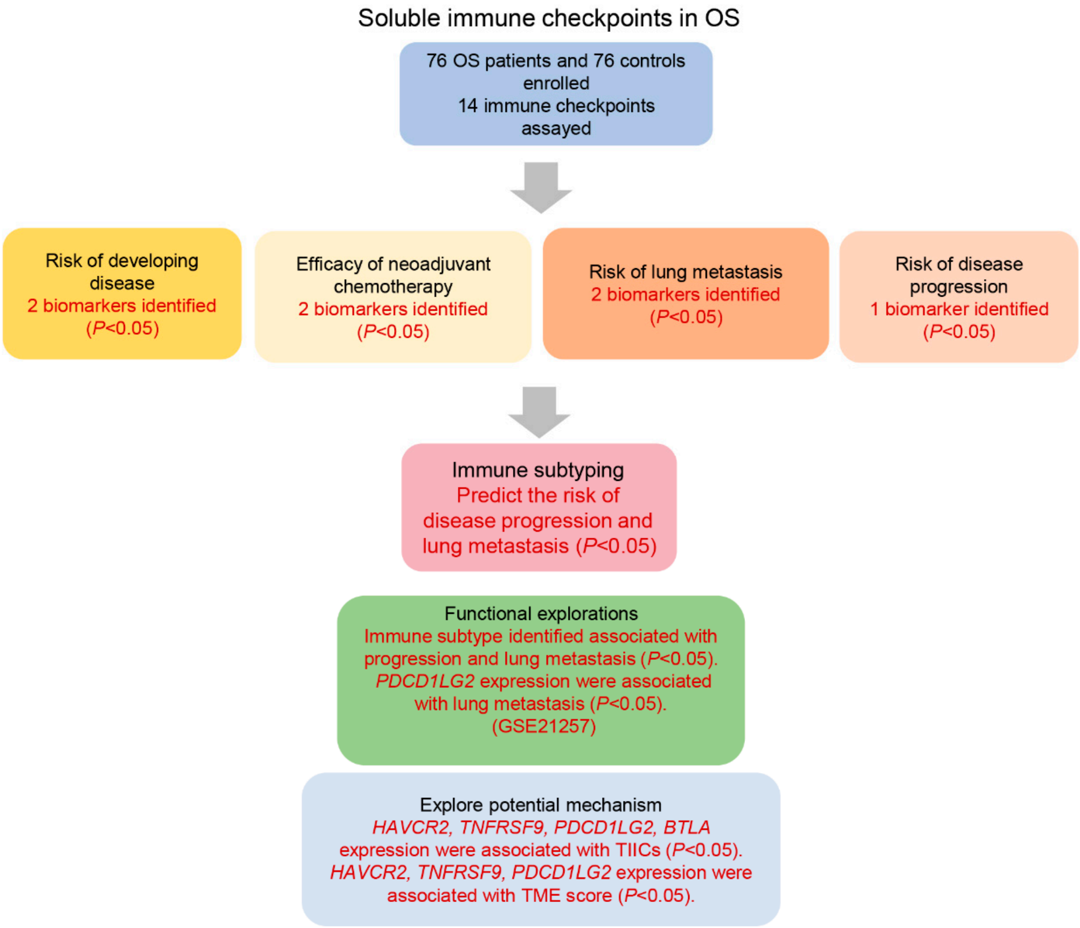

Supplemental Figure S1: A schematic deign of the study.

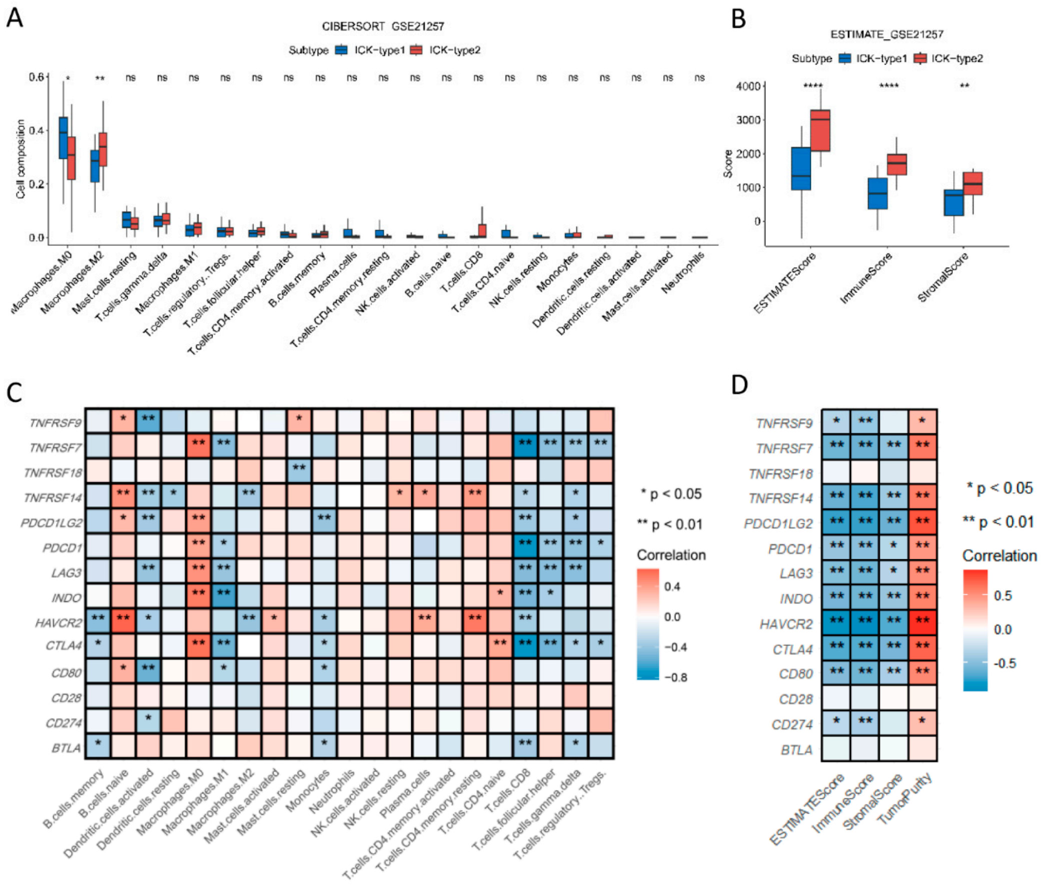

Supplemental Figure S2: Immune landscape of osteosarcoma in dataset GSE21257. **(A)** Assessment of Tumor-Infiltrating Immune Cells (TIICs) in the two immune subtypes. ns≥ 0.05, *<0.05, **<0.01, ***<0.001,****<0.0001. **(B)**. The association of ICK immune subtypes and Tumor Microenvironment (TME) score. Patients in ICK-type2 had significantly higher proportion of M2 macrophages and lower proportion of M0 macrophages than the patients in ICK-type1 *<0.05, **<0.01. **(C-D)** The associations between individual ICK genes and TIIC TME scores were also performed.

Supplemental Tables

Supplemental Table S1. Lower limits of quantification (LLOQ) of each analyte (data from ThermoFisher†).

Analyte	LLOQ (pg/ml)
BTLA	126.39
CD27	4.83
CD28	32.47
CD80	41.82
CD137 (4-1BB)	15.14
CD152 (CTLA4)	8.57
GITR	21.22
HVEM	15.01
IDO	3.66
LAG-3	12.00
PD-1	6.98
PD-L1	3.49
PD-L2	45.00
TIM-3	59.59

† ProcartaPlex Human Immuno-Oncology Checkpoint Panel, https://www.thermofisher.com/order/catalog/product/EPX14A-15803-901

Supplemental Table S2. Peripheral levels of soluble immune checkpoint-related proteins in osteosarcoma patients and controls.

Marker	Osteosarcoma (n=76)	Health control (n=76)	*P*
	Median (IQR) pg/ml	Median (IQR) pg/ml	
sTIM3	477.80 [348.97, 646.44]	364.75 [197.56, 518.27]	**1.86E-03**
sCD28*	32.47 [32.47, 79.77]	32.47 [32.47, 43.92]	-
sCD137	55.25 [38.42, 79.57]	34.69 [15.98, 54.58]	**4.80E-04**
sCD27	82.69 [40.20, 206.43]	62.84 [35.13, 106.82]	0.10
sCTLA4	12.22 [8.86, 15.46]	13.68 [10.32, 22.04]	**2.65E-02**
sIDO	23.48 [12.07, 33.42]	18.72 [12.00, 32.48]	0.66
sLAG3	16.02 [12.00, 21.54]	13.95 [12.00, 28.24]	0.92
sBTLA	326.16 [250.75, 537.74]	341.25 [254.25, 521.35]	0.99
sCD80	43.22 [41.82, 86.72]	64.98 [41.82, 141.72]	0.05
sPD1	37.54 [22.03, 52.12]	40.15 [26.61, 62.90]	0.16
sPDL2	1995.28 [1237.72, 3052.60]	2409.48 [1730.57, 3266.61]	**2.74E-02**
sHVEM*	15.01 [15.01, 15.01]	15.01 [15.01, 15.01]	-
sPDL1*	3.49 [3.49, 3.49]	3.49 [3.49, 3.49]	-
sGITR*	21.22 [21.22, 21.22]	21.22 [21.22, 21.22]	-

*sHVEM, sPDL1, sGITR and sCD28 were not included in the subsequent analysis for minimal variations.

Supplemental Table S3. Univariate Cox Hazards Model of lung metastasis and progression of osteosarcoma.

Markers	Lung Metastasis		Progression	
High vs low[a]	HR (95%CI)	*P* value	HR (95%CI)	*P* value
sTIM3	1 (reference)		1 (reference)	
	2.99 (0.66-13.5)	0.1555	3.22 (0.94-11.08)	0.0630
sCD137	1 (reference)		1 (reference)	
	0.55 (0.15-2.01)	0.3689	0.65 (0.23-1.81)	0.4084
sCD27	1 (reference)		1 (reference)	
	2.70 (0.88-8.27)	0.0816	2.01 (0.81-4.94)	0.1307
sCTLA4	1 (reference)		1 (reference)	
	1.89 (0.62-5.79)	0.2665	0.99 (0.36-2.75)	0.9810
sIDO	1 (reference)		1 (reference)	
	3.26 (0.90-11.85)	0.0727	2.18 (0.83-5.74)	0.1142
sLAG3	1 (reference)		1 (reference)	
	0.44 (0.15-1.31)	0.1385	0.62 (0.25-1.54)	0.3068
sBTLA	1 (reference)		1 (reference)	
	**0.26 (0.09-0.77)**	**0.0153**	**0.36 (0.15-0.90)**	**0.0285**
sCD80	1 (reference)		1 (reference)	
	**0.21 (0.06-0.78)**	**0.0196**	0.57 (0.23-1.42)	0.2278
sPD1	1 (reference)		1 (reference)	
	0.35 (0.11-1.15)	0.0850	**0.38 (0.14-0.99)**	**0.0482**
sPDL2	1 (reference)		1 (reference)	
	**12.08 (1.57-92.94)**	**0.0167**	2.68 (0.96-7.44)	0.0591

Abbreviations: HR Hazard ratio, CI Confidence interval. Significant values in bold font

[a]High and low level groups dichotomized by the R-package survminer

Supplemental Table S4. Peripheral levels of soluble immune checkpoint-related proteins in osteosarcoma patients and controls. levels of soluble immune checkpoint-related proteins in different Huvos grade.

Marker	Huvos grade I (n=3)	Huvos grade II-IV (n=56)	*p*
	Median (IQR) pg/ml	Median (IQR) pg/ml	
sTIM3	195.86 [127.73, 273.73]	477.80 [348.97, 631.72]	**3.40E-02**
sCD137	20.24 [17.69, 39.88]	53.84 [39.35, 80.76]	0.12
sCD27	30.43 [23.45, 68.24]	94.75 [42.90, 226.16]	0.16
sCTLA4	9.00 [8.78, 10.49]	12.96 [9.58, 16.39]	0.14
sIDO	12.54 [8.10, 18.83]	24.23 [14.61, 36.98]	0.16
sLAG3	14.42 [13.21, 24.52]	16.15 [12.00, 21.10]	0.97
sBTLA	166.96 [148.63, 226.23]	312.04 [243.19, 509.71]	0.08
sCD80	41.82 [41.82, 63.52]	41.82 [41.82, 79.25]	0.67
sPD1	17.12 [12.05, 20.61]	38.61 [23.03, 51.85]	**3.70E-02**
sPDL2	1110.37 [925.11, 1712.71]	1828.17 [1304.77, 2666.77]	0.28

Supplemental Table S5. Clinical characteristics of immune subtypes in SAH.

		sICK-type1 (n=35)	sICK-type2 (n=41)	*P*
Gender(%)				0.71
	Male	27 (77.14)	29 (70.73)	
	Female	8 (22.86)	12 (29.27)	
Age(%)				0.04
	<18years	21 (60.00)	14 (34.15)	
	⩾18years	14 (40.00)	27 (65.85)	
Tumor size(%)				0.31
	<8cm	23 (65.71)	20 (48.78)	
	⩾8cm	12 (34.29)	19 (46.34)	
	Missing	0 (0.00)	2 (4.88)	
Tumor location(%)				1.00
	Extremities	30 (85.71)	36 (87.80)	
	Non-extremities	5 (14.29)	5 (12.20)	
Huvos_grade (%)				0.78
	I and II	12 (34.29)	12 (29.27)	
	III and IV	15 (42.86)	20 (48.78)	
	Missing	8 (22.85)	9 (21.95)	
Metastasis (%)				0.01
	Non-metastasis	28 (80.00)	26 (63.41)	
	Baseline_metastasis	6 (17.14)	3 (7.32)	
	followup_metastasis	1 (2.86)	12 (29.27)	
Progression (%)				0.01
	No	32 (91.43)	25 (60.98)	
	Yes	3 (8.57)	16 (39.02)	

Supplemental Table S6. Clinical characteristics of immune subtypes in dataset of GSE21257

		ICK-type1 (n=27)	ICK-type2 (n=26)	*P* value
Gender (%)				0.918
	Male	18 (66.67)	16 (61.54)	
	Female	9 (33.33)	10 (38.46)	
Age (%)				0.918
	<18years	18 (66.67)	16 (61.54)	
	⩾18years	9 (33.33)	10 (38.46)	
Histological (%)				1.000
	Osteoblastic	16 (59.26)	16 (61.54)	
	Others	11 (40.74)	10 (38.46)	
Tumor location (%)				0.248
	Extremities	20 (74.07)	24 (92.31)	
	Non-extremities	6 (22.22)	2 (7.69)	
	Missing	1 (3.71)	0 (0.00)	
Huvos grade (%)				0.166
	I and II	17 (62.96)	12 (46.15)	
	III and IV	6 (22.22)	12 (46.15)	
	Missing	4 (14.82)	2 (7.70)	
Metastasis (%)				0.003
	Non-metastasis	4 (14.81)	15 (57.69)	
	Baseline metastasis	15 (55.56)	5 (19.23)	
	Follow-up metastasis	8 (29.63)	6 (23.08)	
Progression (%)				0.009
	No	7 (25.93)	17 (65.38)	
	Yes	20 (74.07)	9 (34.62)	
